# The relationship between urinary Alzheimer-associated neuronal thread protein and blood biochemical indicators in the general population

**DOI:** 10.18632/aging.103356

**Published:** 2020-07-31

**Authors:** Yuxia Li, Shaochen Guan, He Jin, Hongjun Liu, Meimei Kang, Xiaozhen Wang, Can Sheng, Yu Sun, Xuanyu Li, Xianghua Fang, Rong Wang

**Affiliations:** 1 Central Laboratory, Xuanwu Hospital, Capital Medical University, Beijing 100053, China; 2 Evidence-Based Medical Center, Xuanwu Hospital, Capital Medical University, Beijing 100053, China; 3 Beijing Geriatric Medical Research Center, Beijing 100053, China; 4 Clinical Laboratory, Affiliated Hospital of Guilin Medical University, Guilin 541001, China; 5 Department of Neurology, Xuanwu Hospital of Capital Medical University, Beijing 100053, China; 6 Center of Alzheimer’s Disease, Beijing Institute for Brain Disorders, Beijing 100053, China

**Keywords:** Alzheimer’s disease, Alzheimer-associated neuronal thread protein, urine, serum creatinine

## Abstract

Urinary Alzheimer-associated neuronal thread protein (AD7c-NTP) is elevated in early Alzheimer's disease (AD) and mild cognitive impairment, and is considered a biomarker for the early diagnosis of AD. However, it has not yet been investigated whether urinary AD7c-NTP is elevated with increases in blood biochemical indicators related to AD risk factors. We recruited 2180 participants, aged 35–93 years, from communities of four districts in Beijing. Blood biochemical indicators, including blood glucose, blood lipids, renal function, and high-sensitivity C-reactive protein, were measured using routine methods. Urinary AD7c-NTP was detected using an enzyme-linked immunosorbent assay AD7c-NTP kit. In the general population, there were no significant differences in urinary AD7c-NTP levels in subjects with different Mini–Mental State Examination levels or C-reactive protein values. After adjusting for age and sex, there were significant differences in urinary AD7c-NTP levels between different education levels, marital statuses, blood glucose, blood lipids, and kidney function. There was a negative correlation between urinary AD7c-NTP levels and serum creatinine (*r* = –0.128). There was a positive correlation between urinary AD7c-NTP levels and HbA1c (*r* = 0.104), insulin (*r* = 0.101), and triglycerides (*r* = 0.093). Urinary AD7c-NTP might be useful as a potential indicator to predict AD risk.

## INTRODUCTION

Alzheimer's disease (AD) is characterized by memory loss, decreased verbal and logical thinking abilities, and impairments in other cognitive domains. It is the most common cause of dementia, accounting for 60%–80% of all cases [1, 2]. AD is a disabling, irreversible neurodegenerative disease that is costly to treat and places a heavy burden on society and families [3, 4]. The early diagnosis of AD is of great significance for the prevention and treatment of this dementia. In 2011, the concept of biomarkers was introduced into the diagnostic criteria for AD that is published by the National Institute on Aging-Alzheimer's Association [5–7]. Biomarker evidence may enhance the pathophysiological specificity of an AD diagnosis. Thus, biomarkers that are stable, not easily influenced by other factors, convenient, fast, safe, non-invasive, non-radiative, and reproducible, are urgently needed to improve the early diagnosis of AD.

Alzheimer-associated neuronal thread protein (AD7c-NTP) is a type of neuronal thread protein that is closely related to the pathophysiological mechanisms of AD [8, 9]. Previous studies have shown that AD7c-NTP immunoreactivity co-localizes with tau-immunoreactive neurofibrillary tangles and malnourished neurites, and that there is abnormal AD7c-NTP gene expression before the formation of neurofibrillary tangles [9]. Several studies have confirmed that AD7c-NTP can be detected in the early stage of AD in cortical neurons, brain tissue extracts, cerebrospinal fluid (CSF), and urine. Additionally, AD7c-NTP in urine has a similar specificity and sensitivity for AD diagnosis as it does in CSF [10–13]. Urinary AD7c-NTP is elevated in early AD and mild cognitive impairment (MCI) and is considered to be a biomarker for the early diagnosis of AD [14–18]. Studies over the past two decades have revealed that in the early stage of AD, urinary AD7c-NTP levels are positively correlated with AD severity; that is, when cognitive levels are lower, urinary AD7c-NTP levels are higher [19–21].

Urinary AD7c-NTP may be a promising biomarker for the early diagnosis of AD because of its advantages of being non-invasive, non-radiative, simple to measure, and highly repeatable [17]. Urine is susceptible to many physiological and pathological factors, as well as other confounding factors [22]. Therefore, an investigation into factors that might interfere with urinary AD7c-NTP testing is needed, using data from a large sample. Studies have shown that urinary AD7c-NTP tends to increase with age and that it is slightly higher in females than in males [23, 24]. It has also been reported that urinary AD7c-NTP levels are not affected by demographic factors such as years of education, employment status, current place of residence, body mass index, or the presence of common chronic diseases such as hypertension, diabetes, stroke, dyslipidemia, renal insufficiency, cancer, chronic lung disease, chronic liver disease, or depression [19, 23, 25]. However, the relationship between renal function, blood glucose, blood lipids, inflammation indicators, and urinary AD7c-NTP levels has not been studied in a large sample. Thus, the purpose of this epidemiological study was to investigate whether urinary AD7c-NTP levels were elevated with increased renal function, blood glucose, blood lipids, inflammation indicators, or other demographic and clinical characteristics in the general population.

## RESULTS

### Demographic and clinical characteristics of participants

In this study, 2180 participants aged 35-93 years old living in four districts in Beijing were recruited in the general population from the community. Participants had an average age of 64.94 ± 9.88 years, and 42.5% (*n* = 927) were male. Because of the large number of participants in this epidemiological investigation, some participants' information was not collected or registered during the study. We collected data about years of education from 2161 participants, Mini–Mental State Examination (MMSE) scores from 1495 participants, and marital status from 2137 participants. For clinical data, we measured serum creatinine (CR), total cholesterol (TC), triglyceride (TG), and high-density lipoprotein cholesterol (HDL-C) levels in 2155 participants, urinary microalbumin (UMA) levels in 1581 participants, blood glucose (GLU) and low-density lipoprotein cholesterol (LDL-C) levels in 1706 participants, glycated hemoglobin A1c (HbA1c) levels in 1839 participants, insulin levels in 1307 participants, high-sensitivity C-reactive protein (hsCRP) levels in 1256 participants, and uric acid (UA) levels in 1844 participants. We calculated the distribution of the blood biochemical indicators and urinary AD7c- NTP and made the histograms, shown as in the Supplementary material (Supplementary Figures 5–16). The comparisons of essential demographic and clinical characteristics between different groups are shown in Table 1. Blood biochemical indicator values are listed in Table 2.

**Table 1:** Demographic and clinical characteristics of study participants.

**Variables**	**n**	**Subject group**	**Age (years)**	**Male: female**	**Number**	**Percent (%)**
Age, years	2180	All	66.94±9.88	927:1253	2180	100.0
		<60	50.76±6.30	177:241	418	19.2
		60-69	65.76±2.56	325:503	828	38.0
		70-79	73.96±2.80	354:438	792	36.3
		>80	82.36±2.62	71:71	142	6.5
Sex	2180	male	67.32±10.02	100:0	927	42.5
		female	66.67±9.76	0:100	1253	57.5
Education level	2161	<12	68.05±9.10	632:941	1573	72.2
		=12	64.12±10.46	180:225	405	18.6
		>12	64.36±11.80	106:77	183	8.4
MMSE	1495	normal	71.19±5.03	527:768	1295	59.4
		abnormal	74.49±6.17	102:98	200	9.2
Spouse	2137	with spouse	65.90±9.75	816:976	1792	82.2
		without spouse	72.59±7.92	93:252	345	15.8
CR	2155	normal	66.38±9.95	788:1060	1848	85.8
		reduced	69.61±7.35	42:82	124	5.7
		elevated	71.10±9.66	89:94	183	8.5
UMA	1581	normal	65.00±10.42	501:645	1146	72.5
		abnormal	67.22±10.49	172:263	435	27.5
UA	1844	normal	67.96±9.05	658:894	1552	84.2
		reduced	66.14±10.61	17:88	105	5.7
		elevated	70.03±9.42	119:68	187	10.1
GLU	1706	normal	64.47±11.27	371:580	951	55.7
		reduced	71.83±5.14	26:63	89	5.2
		elevated	66.98±9.28	314:352	666	39.0
HbA1c	1839	normal	67.44±9.93	557:691	1248	67.8
		elevated	69.40±7.35	236:355	591	32.1
insulin	1307	normal	66.66±10.10	513:713	1226	93.8
		reduced	68.29±9.34	33:12	45	3.4
		elevated	66.42±8.36	14:22	36	2.8
TC	2155	normal	67.32±9.89	688:685	1373	63.7
		abnormal	66.35±9.88	231:551	782	36.3
TG	2155	normal	67.02±9.97	680:836	1516	70.3
		abnormal	66.85±9.73	239:400	639	29.7
LDL-C	1706	normal	66.11±10.61	512:586	1098	64.4
		abnormal	65.34±10.13	199:409	608	35.6
HDL-C	2155	normal	66.83±9.92	807:1180	1987	92.2
		abnormal	68.61±9.50	112:56	168	7.8
hsCRP	1256	normal	66.86±9.74	484:640	1124	89.5
		abnormal	67.99±10.00	53:79	132	10.5

Abbreviation: MMSE = Mini–Mental State Examination; CR = serum creatinine; UMA = urinary microalbumin; UA = uric acid; GLU = glucose; HbA1c = hemoglobin A1c; TC = total cholesterol; TG = triglycerides; LDL-C = low-density lipoprotein cholesterol; HDL-C = high-density lipoprotein cholesterol; hsCRP = hypersensitive C-reactive protein.

**Table 2:** Distribution and grouping the cut-off value of blood biochemical indicators of study participants.

**Variables**	**n**	**median (IQR)**	**normal cut-off value**	**abnormal group**	**reduced group**	**elevated group**	**skewness**	**kurtosis**
CR (μmol/L)	2155	82.000(23.000)	62-115 (male); 53-97 (female)		<62 <53	>115 (male); >97 (female)	2.901±0.053	25.827±0.105
UMA (mg/l)	1581	9.880(15.300)	<20	≥20			13.194±0.062	229.036±0.123
UA (μmol/L)	1844	307.550(105.500)	200-420 (male); 140-340(female)		<200 <140	>420 (male); >340 (female)	0.7430±0.057	1.400±0.114
GLU (mmol/l)	1706	5.800(1.190)	3.9-6.1		<3.9	>6.1	1.506±0.590	8.866±0.118
HbA1c (%)	1839	5.700(0.900)	4.0-6.0%		<4.0	>6.0	2.432±0.057	8.594±0.114
insulin (nU/ml)	1307	8.510(7.010)	2.6-24.9		<2.6	>24.9	8.563±0.068	113.584±0.135
TC (mmol/l)	2155	4.860(1.370)	<5.2	≥5.2			0.761±0.053	2.855±0.105
TG (mmol/l)	2155	1.260(0.930)	<1.7	≥1.7			6.337±0.053	74.777±0.105
LDL-C (mmol/l)	1706	2.779(1.190)	<3.12	≥3.12			0.412±0.059	0.877±0.118
HDL-C (mmol/l)	2155	1.290(0.450)	>0.91	≤0.91			1.312±0.503	5.290±0.125
hsCRP (mg/l)	1256	0.120(0.190)	<0.5	≥0.5			4.821±0.069	33.230±0.138

Abbreviation: CR = serum creatinine; UMA = urinary microalbumin; UA = uric acid; GLU = glucose; HbA1c = hemoglobin A1c; TC = total cholesterol; TG = triglycerides; LDL-C = low-density lipoprotein cholesterol; HDL-C = high-density lipoprotein cholesterol; hsCRP = hypersensitive C-reactive protein; IQR = interquartile range.

### Urinary AD7c-NTP levels by sex and age

We calculated the distribution of urinary AD7c-NTP in different age groups and made histograms, as shown in the Supplementary material (Supplementary Figures 1–4). As shown in Table 3, urinary AD7c-NTP levels in female subjects [0.2241 (0.2964) ng/mL] were signify-cantly higher than in male subjects [0.1613 (0.2133) ng/mL; *p* < 0.001]. Table 3 also shows that urinary AD7c-NTP levels were significantly different among the <60, 60–69, 70–79, and 80–89 years-old groups (*p* < 0.05). Furthermore, we found that urinary AD7c-NTP levels were lower in the <60 years group than in the 60–69 years group. There were significant differences between the oldest group (80–89 years) and the other three groups (<60, 60–69, and 70–79 years). Urinary AD7c-NTP levels in the oldest group [80–89 years; 0.2126 (0.3152) ng/mL] were higher than those in the other three groups [<60 years, 0.1695 (0.1699) ng/mL; 60–69 years, 0.1998 (0.2667) ng/mL; 70–79 years, 0.2033 (0.2967) ng/mL]. After adjusting for age and sex, the results were consistent with the previous findings that urinary AD7c-NTP levels were signifycantly higher in females and with age (*p* < 0.001, Table 3).

**Table 3:** Urinary AD7c-NTP levels grouped according to demographic characteristics and blood biochemical indicators.

**Variables**	**Subject group**	**AD7c-NTP(ng/ml)**	***Z/χ2***	***p***	**Adjusted AD7c-NTP^†^(ng/ml)**	***Z/χ2***	***p***
Sex	male	0.1613 (0.2133)	-8.417	<0.001*	0.1586 (0.0124)	-39.924	<0.001*
	female	0.2241 (0.2964)			0.2264 (0.0174)		
Age, y	All	0.1936 (0.2569)			0.1976 (0.0369)		
	<60	0.1695 (0.1699)	14.387	0.002*	0.1732 (0.0310)	433.417	<0.001*
	60-69	0.1998 (0.2667)			0.1974 (0.0335)		
	70-79	0.2033 (0.2967)			0.2070 (0.0364)		
	>80	0.2126 (0.3152)			0.2175 (0.0392)		
Education level	<12	0.1962 (0.2571)	4.763	0.092	0.2010 (0.0368)	51.954	<0.001*
	=12	0.1839 (0.2404)			0.1917 (0.0354)		
	>12	0.1961 (0.2862)			0.1829 (0.0347)		
MMSE	abnormal	0.2157 (0.2842)	-0.405	0.686	0.2053 (0.0354)	-0.1466	0.143
	normal	0.2039 (0.2767)			0.2033 (0.0369)		
Spouse	with spouse	0.1907 (0.2603)	-0.579	0.563	0.1937 (0.0358)	-12.480	<0.001*
	without spouse	0.2016 (0.2382)			0.2180 (0.0351)		
CR	normal	0.1866 (0.2486)	8.861	0.012*	0.1966 (0.0369)	13.393	0.001*
	reduced	0.2639 (0.3678)			0.2077 (0.0347)		
	elevated	0.2143 (0.3038)			0.1997 (0.0371)		
UMA	normal	0.1805 (0.2536)	-1.405	0.160	0.1938 (0.0369)	-3.385	0.001*
	elevated	0.1632 (0.2172)			0.2001 (0.0368)		
UA	normal	0.1975 (0.2536)	2.212	0.331	0.1992 (0.0367)	25.740	<0.001*
	reduced	0.1628 (0.2954)			0.2144 (0.0307)		
	elevated	0.2079 (0.2457)			0.1879 (0.0377)		
GLU	normal	0.1756 (0.2543)	1.212	0.546	0.1962 (0.0368)	27.419	<0.001*
	reduced	0.1572 (0.2150)			0.2147 (0.0342)		
	elevated	0.1763 (0.2264)			0.1945 (0.0373)		
HbA1c	normal	0.1846 (0.2471)	-3.957	<0.001*	0.1969 (0.0371)	-2.976	0.003*
	elevated	0.2295 (0.2858)			0.2031 (0.0358)		
Insulin	normal	0.1789 (0.2462)	8.075	0.018*	0.1976 (0.0370)	9.503	0.009*
	reduced	0.1999 (0.1770)			0.1787 (0.0362)		
	elevated	0.2174 (0.2964)			0.1989 (0.0350)		
TC	normal	0.1873 (0.2616)	-1.467	0.142	0.1929 (0.0370)	-7.033	<0.001*
	elevated	0.1999 (0.2439)			0.2057 (0.0353)		
TG	normal	0.1816 (0.2565)	-4.077	<0.001*	0.1960 (0.0365)	-2.774	0.006*
	elevated	0.2163 (0.2534)			0.2012 (0.0376)		
LDL-C	normal	0.1728 (0.2507)	-0.347	0.729	0.1935 (0.0373)	-3.891	<0.001*
	elevated	0.1802 (0.2367)			0.2019 (0.0362)		
HDL-C	normal	0.1930 (0.2537)	-0.099	0.921	0.1987 (0.0369)	-4.692	<0.001*
	reduced	0.1963 (0.2880)			0.1831 (0.0337)		
hsCRP	normal	0.1795 (0.2450)	-0.385	0.700	0.1971 (0.0368)	-1.232	0.218
	elevated	0.1622 (0.2628)			0.2011 (0.0382)		

†Adjusted for age and sex.

Abbreviation: MMSE = Mini–Mental State Examination; CR = serum creatinine; UMA = urinary microalbumin; UA = uric acid; GLU = glucose; HbA1c = hemoglobin A1c; TC = total cholesterol; TG = triglycerides; LDL-C = low-density lipoprotein cholesterol; HDL-C = high-density lipoprotein cholesterol; hsCRP = hypersensitive C-reactive protein. The asterisk indicates a *p* value less than 0.05. *Z* refers to the statistical value of comparison between groups using Mann-Whitney U test.

### Urinary AD7c-NTP levels by education level, marital status, and MMSE scores

There were no differences in urinary AD7c-NTP levels between different education levels or marital statuses (Table 3; *p* > 0.05). In addition, there were no significant differences in urinary AD7c-NTP levels between the normal MMSE group and the abnormal MMSE group (*p* > 0.05) in our study.

After adjusting for age and sex, there were significant differences in urinary AD7c-NTP levels between different education levels or marital statuses (Table 3). Urinary AD7c-NTP levels were slightly increased in people with lower education and those without a spouse.

### Correlation analyses between urinary AD7c-NTP levels and blood biochemical indicators

There was no significant correlation between urinary AD7c-NTP levels and UMA, UA, GLU, TC, HDL-C, LDL-C, or hsCRP levels. However, there was a negative correlation between urinary AD7c-NTP levels and CR values (r=-0.128, Table 4). There was a positive correlation between urinary AD7c-NTP levels and HbA1c (*r*=0.104), insulin (r=0.101) and TG (r=0.093).

**Table 4:** Correlation of urinary AD7c-NTP levels and blood biochemical indicators.

	**AD7c-NTP (ng/ml)**	**r**	**p**
CR	0.1930 (0.2559)	-0.128	<0.001*
UMA	0.1756 (0.2498)	0.004	0.866
UA	0.1970 (0.2557)	-0.003	0.906
GLU	0.1751 (0.2461)	0.008	0.748
HbA1c	0.1970 (0.2560)	0.104	<0.001*
insulin	0.1779 (0.2459)	0.101	<0.001*
TC	0.1930 (0.2559)	0.023	0.277
TG	0.1930 (0.2559)	0.093	<0.001*
LDL-C	0.1751 (0.2461)	0.022	0.357
HDL-C	0.1930 (0.2559)	-0.042	0.053
hsCRP	0.1768 (0.2467)	0.049	0.084

Abbreviation: CR = serum creatinine; UMA = urinary microalbumin; UA = uric acid; GLU = glucose; HbA1c = hemoglobin A1c; TC = total cholesterol; TG = triglycerides; LDL-C = low-density lipoprotein cholesterol; HDL-C = high-density lipoprotein cholesterol; hsCRP = hypersensitive C-reactive protein. The asterisk indicates a p value less than 0.05.

### The association between urinary AD7c-NTP levels and blood biochemical indicator values

According to the Mann-Whitney U test, there were no significant differences in urinary AD7c-NTP levels in subjects with different GLU, UA, UMA, TC, LDL-C, HDL-C, or hsCRP values.

In Table 3, it is notable that the most significant differences (*p* =0.012) in urinary AD7c-NTP levels occur among the CR normal, CR reduced, and CR elevated groups. Further comparisons of the three groups we found that urinary AD7c-NTP levels were significantly higher in the CR reduced group [0.2639 (0.3678) ng/mL] than those in the CR normal group [0.1866 (0.2486) ng/mL], and that urinary AD7c-NTP levels were significantly higher in the CR reduced group [0.2639 (0.3678) ng/mL] compared with the CR elevated group [0.2143 (0.3038) ng/mL].

Furthermore, there were significant differences in urinary AD7c-NTP levels in subjects with different HbA1c (*p* < 0.001), insulin (*p* = 0.018) and TG (*p* < 0.001). Further comparisons showed that urinary AD7c-NTP levels were significantly higher in the HbA1c elevated group [0.2295 (0.2858) ng/mL] than those in the HbA1c normal group [0.1846 (0.2471) ng/mL]. Urinary AD7c-NTP levels were significantly higher in the insulin elevated group [0.2174 (0.2964) ng/mL] than those in the insulin normal group [0.1789 (0.2462) ng/mL] and insulin reduced group [0.1999 (0.1770) ng/mL]. Urinary AD7c-NTP levels were significantly higher in the TG abnormal group [0.2163 (0.2534) ng/mL] than those in the TG normal group [0.1816 (0.2565) ng/mL].

After adjusting for age and sex, there were significant differences in urinary AD7c-NTP levels between different GLU, HbA1c, insulin, CR, UA, UMA, TC, TG, LDL-C, and HDL-C values. Urinary AD7c-NTP levels were significantly higher in the CR-reduced, UMA-elevated, UA-reduced, GLU-reduced, HbA1c- a elevated, insulin-elevated, TC-elevated, TG-elevated, LDL-C-elevated, and HDL-C-reduced groups (Table 3). After adjusting for age and sex, there was no significant difference in adjusted urinary AD7c-NTP levels in subjects with different C-reactive protein (hsCRP) values.

## DISCUSSION

In this study, we investigated the relationship between demographic characteristics, clinical characteristics, blood biochemical indicators, and urinary AD7c-NTP in a large sample as an epidemiological investigation of the general population. Our study found that i) urinary AD7c-NTP increased slightly in females and with age in the general population, and was slightly higher in people with lower education and those without a spouse; ii) there were significant differences in urinary AD7c-NTP levels in subjects with different GLU, blood lipids, and kidney function; iii) there was a negative correlation between urinary AD7c-NTP levels and CR values; and iv) there was a positive correlation between urinary AD7c-NTP levels and HbA1c, insulin, and TG values. The results also suggest that, in the general population, urinary AD7c-NTP levels are not elevated with increased hsCRP. In subjects with the AD risk factors of elevated HbA1c, insulin, TC, TG, and LDL-C, as well as reduced HDL-C, urinary AD7c-NTP was slightly increased but did not reach the cut-off value to distinguish the diagnosis of AD dementia.

### The relationship between urinary AD7c-NTP levels and age and sex

In our study, urinary AD7c-NTP tended to increase with age and was slightly higher in females than in males, which was consistent with previous research [23, 24]. Urinary AD7c-NTP levels in the oldest group of participants were slightly higher than in the other three groups. Urinary AD7c-NTP levels in the 60–69-year-old group were slightly higher than those in participants under 60 years of age. This phenomenon is consistent with previous observations that AD incidence increases with age, and that AD incidence is higher in females than in males [26, 27]. It is well known that age is the most influential risk factor for AD dementia; the incidence of AD and MCI increases exponentially with increasing age [28, 29]. Existing research has recognized the critical role played by age in AD development. In addition, several studies have analyzed the possible reasons why women are more likely to have AD dementia than men: for example, women may be more susceptible to the adverse effects of head injuries, women have fewer opportunities for education than men, women have a higher risk of depression than men, women have longer life expectancies than men, and sex steroids can affect cognitive function [27, 30]. Therefore, the establishment of urinary AD7c-NTP thresholds for different age groups and sexes is still needed.

### The relationship between urinary AD7c-NTP levels and MMSE, education level, and marital status

In recent years, the correlation between urinary AD7c-NTP and cognitive levels has received increasing attention from researchers. Previous research into the relationship between urinary AD7c-NTP levels and MMSE has been controversial. Some studies have reported a negative correlation between urinary AD7c-NTP levels and MMSE [19, 20], while other studies have not found such a relationship [14]. In our epidemiological study of the general population, urinary AD7c-NTP appeared to be slightly increased in the MMSE abnormal group, but this difference was not statistically significant. A possible reason for this phenomenon is that only 1495 of the 2180 subjects completed the MMSE scale examination, and there were only 200 subjects in the MMSE abnormal group (accounting for 9.2% of the total sample population). The majority of people who did not complete the MMSE scale were cognitively normal and did not need to be examined. The increases in urinary AD7c-NTP levels in the MMSE abnormal group was not found in the general population with normal cognition. Therefore, future studies should use larger sample sizes and include more participants with abnormal MMSE scores. In this way, any possible relationship between urinary AD7c-NTP and MMSE levels can be accurately examined.

Low education has long been associated with the risk of AD [31]. In addition, AD patients who have their spouses as their caregivers have a relatively slow cognitive decline, and self-rated quality of life scores are higher in AD patients who live with their spouses [32]. In our study, after adjusting for age and sex, there were significant differences in urinary AD7c-NTP levels between different education levels and marital statuses. These results suggest that urinary AD7c-NTP is increased in the lower-educated and unmarried population, which has a higher risk of AD.

### The relationship between urinary AD7c-NTP levels and blood glucose and hsCRP

Urine is a waste product and toxin expelled from the body. AD7c-NTP appears in the urine through blood filtration; fluctuations in urine are more significant than those in blood and better reflect changes in the body [33]. It was previously unclear whether urinary AD7c-NTP is elevated with increases in blood glucose, blood lipids, kidney function, or inflammation. In this study, we analyzed the correlation between urinary AD7c-NTP and blood lipids (TC, TG, LDL-C, HDL-C), blood glucose (GLU, insulin, HbA1c), renal function (CR, UMA, UA), and biochemical indicators related to an inflammatory response (hsCRP). There was no significant difference in urinary AD7c-NTP levels in subjects with different hsCRP levels, as shown in Table 3. However, there were significant differences in urinary AD7c-NTP levels in subjects with different GLU, HbA1c, and insulin levels.

Recently, it has become well established that modifiable vascular risk factors are essential for AD risk [34–36]. Previous studies have shown that about one-third of AD dementia can be attributed to modifiable vascular risk factors [37]. Current research suggests that type 2 diabetes mellitus and hyperinsulinemia increase the risk for AD, possibly through their effects on amyloid-beta metabolism and cerebrovascular dysfunction [38]. In our study, urinary AD7c-NTP levels were slightly higher in subjects with higher HbA1c or insulin levels. In addition, correlation analyses revealed a positive correlation between urinary AD7c-NTP and HbA1c and insulin levels. Because diabetes mellitus and hyperinsulinemia are risk factors for AD, and patients with increased HbA1c and hyperinsulinemia had increased urinary AD7c-NTP in our study, we speculate that people with risk factors for AD may have AD-related pathological changes earlier than people without risk factors for AD. Unexpectedly, however, there was no positive correlation between GLU and urinary AD7c-NTP levels in our study. We speculate that this may be because HbA1c reflects the blood glucose changes in diabetic patients better than fasting GLU levels. The mechanisms of brain inflammation in AD pathogenesis have also attracted more and more attention recently [39]. Further research is therefore needed to explore the relationship between inflammatory factors, blood glucose, and urinary AD7c-NTP.

### Correlation analysis between urinary AD7c-NTP and kidney function

There were significant differences in urinary AD7c-NTP levels in subjects with different UA and UMA after adjusting for age and sex. UMA is an indicator of renal function, but no previous studies have investigated the relationship between UMA and dementia. There was also a difference in urinary AD7c-NTP levels between the abnormal and normal CR groups in our study. Further analysis indicated that the urinary AD7c-NTP levels were higher in the decreased CR group than in the normal and elevated CR groups. These results suggest that the increase in urinary AD7c-NTP is not caused by impaired renal function or increased creatinine. This finding is consistent with results from previous studies [23]. Jin et al. reported no difference in urinary AD7c-NTP levels among subjects with depression, hypertension, stroke, diabetes, dyslipidemia, and renal insufficiency [23]. Another study demonstrated that urinary AD7c-NTP is more accurate when urine creatinine is 50–225 mg/dL [40]. When urine creatinine is less than 50 mg/dL, excessive urinary dilution may result in false negatives, and when urinary creatinine is greater than 225 mg/dL, it can lead to false-positive results because of the concentrations of non-specific excess solutes [40]. The correlation analysis revealed a negative correlation between urinary AD7c-NTP levels and CR values; that is, urinary AD7c-NTP did not increase with increased CR levels. Therefore, the results of this study can only show that urinary AD7c-NTP did not increase because of renal function impairment. However, there are very few previous studies on this topic, and our results need to be further validated with multi-center, clinical experimental data from a large sample.

More recently, literature has emerged that offers contradictory findings of a relationship between UA and AD. Some studies report that UA has antioxidant properties that prevent AD from occurring, and state that UA reduction is a risk factor for AD [41]. Other studies have reported that the occurrence of dementia is related to an increase in UA from the baseline [42]. However, further studies have demonstrated that elevated UA is associated with vascular dementia more than with AD [42]. In the present study, after adjusting for age and sex, urinary AD7c-NTP was slightly higher in the reduced UA group than in the normal and elevated UA groups. Together, these results suggest that there is an increase in urinary AD7c-NTP in the AD risk factor group.

### Correlation analysis between urinary AD7c-NTP and blood lipids

It is generally accepted that the risk of MCI and AD dementia is associated with high TC and LDL-C levels and low HDL-C levels [43]. Previous studies have reported that HDL-C levels are inversely associated with incident AD risk, but the finding is unlikely to be of clinical relevance [44]. In our study, there was a significant difference in urinary AD7c-NTP levels between the different TC, TG, LDL-C, and HDL-C groups. Further correlation analyses revealed a very weak positive correlation between urinary AD7c-NTP and TG levels (*r* = 0.093). This result was statistically significant, but it is unlikely to be of clinical significance because the *r-*value is low. Together, these results suggest that, in the cognitively normal population, urinary AD7c-NTP is increased in people with dyslipidemia, which is a risk factor for AD.

In the current study, there were significant differences in urinary AD7c-NTP levels in subjects with different GLU, blood lipids, and kidney function. Further correlation analysis revealed a positive correlation between urinary AD7c-NTP and HbA1c, insulin, and TG levels. However, the correlation coefficients were very low (approximately 0.1). In addition, urinary AD7c-NTP did not increase with increasing hsCRP values, suggesting that urinary AD7c-NTP is a relatively stable indicator that may accurately reflect disease mechanisms without interference from other blood components. Furthermore, urinary AD7c-NTP was positively correlated with HbA1c, insulin, and TG, suggesting that urinary AD7c-NTP levels were slightly higher in subjects with risk factors for AD. Urinary AD7c-NTP might therefore be used as a potential indicator to predict the risk of AD.

### Limitations of the study

There are many limitations to our study. First, the neuropsychological assessment was relatively superficial in our epidemiological survey data; we only used the MMSE. If we combined this examination with the Montreal Cognitive Assessment, Auditory Verbal Learning Test, and other neuropsychological assessment scales, the patients’ cognitive functions would be more comprehensively evaluated. Second, because we conducted a cross-sectional study, any observation of the dynamic relationship between urinary AD7c-NTP levels and blood biochemical indicators was limited. Third, the blood biochemical indicators we selected were not comprehensive; other indicators such as electrolytes and homocysteine should also be determined. Fourth, in this epidemiological study, our data collection was incomplete and there was a lot of missing data. Future studies should expand the sample size and avoid missing data and adopt a multi-center study method to conduct longitudinal studies so that the relationship between urinary AD7c-NTP and blood biochemical indicators can be confirmed.

## CONCLUSION

In view of its tendency to increase with age, urinary AD7c-NTP is a kind of aging-related protein. Urinary AD7c-NTP is a relatively stable and reliable indicator and is not elevated with increased hsCRP levels. After adjusting for age and sex, there were significant differences in urinary AD7c-NTP levels between different education levels, marital statuses, GLU, blood lipids, and kidney function. In subjects with AD risk factors, urinary AD7c-NTP was slightly increased but did not reach the cut-off value. Because urinary AD7c-NTP is increased in subjects with AD risk factors, urinary AD7c-NTP might be useful as a potential indicator to predict the risk of AD. It may also be a promising and feasible biomarker of AD.

## MATERIALS AND METHODS

### Participants

This epidemiological survey study was conducted between August 2014 and December 2015 in Beijing, China. All participants were informed of the procedure at the time of recruitment and were asked to sign an informed consent form before the survey was conducted. This study was approved by the ethics committee of the Xuanwu Hospital of Capital Medical University. A total of 2180 subjects participated in the epidemiological questionnaire, blood sampling for biochemical indicators, and the urinary AD7c-NTP test. Participants aged 35–93 years (*n* = 2180) were recruited and assessed from the general population in the Xi Cheng, Fang Shan, Tong Zhou, and Yan Qing districts in Beijing. To ensure adequate representation of the total population in the epidemiological survey, we adopted a four-stage stratified random procedure using the probability proportional to size method in the sampling process. The details of the technique and process that was used have been described in other articles from our group [23, 45, 46].

### Face-to-face interviews

All participants were asked to complete a standardized questionnaire conducted by trained medical postgraduate students. The questionnaire included questions about demographic information (sex, age, education level, and marital status), menstrual and reproductive history, past medical history, and medication history. General cognitive levels were assessed using the MMSE [47].

### Physical examinations

After the interview, all subjects underwent a physical and clinical examination, including measurements of blood pressure, pulse rate, height, weight, and waist circumference. All evaluations and tests were performed at the local community health clinic or hospital. Each participant was required to rest for at least 20 minutes before the physical examination. Blood pressure and pulse rate were measured three times on the right arm at 5- to 10-minute intervals by trained nurses or medical students, and the means of the three measurements were calculated. Blood pressure was measured using a standard mercury sphygmomanometer while the patient was seated.

### Blood biochemical laboratory measurements

All blood samples were collected from subjects in the morning, following fasting and no water for at least eight hours. We collected approximately 12 mL of blood from each subject using 3 BD tubes, followed by rapid centrifugation of the blood sample using a centrifuge. The supernatant was then stored at 4°C before being transferred to the laboratory. All analytical tests were completed by 24 h post-collection. All laboratory measurements, including GLU, HbA1c, insulin, TC, TG, LDL-C, HDL-C, CR, UMA, UA, and hsCRP were measured using routine methods at Fuwai Hospital, National Center for Cardiovascular Disease. Specifically, TC and TG were assayed using enzymatic methods, while UA was determined by an enzymatic uricase method. CR was determined by an enzymatic assay that was calibrated to the isotope dilution mass spectrometry-traceable creatinine assays. HDL-C was determined using the chemical precipitation method, and UMA concentration was assayed by standard methods using a turbidimetric method. LDL-C was estimated using the Friedwald formula: LDL-C = TC – (HDL-C + TG/2.2) mmol/L. HbA1c was measured using a Biorad Diomat high-pressure liquid chromatography analyzer. A Hitachi 7600 automatic analyzer (Boehringer Mannheim, Mannheim, Germany) was used to determine all biochemical analyses.

### Urinary AD7c-NTP laboratory measurements

Clean midstream urine samples were collected from all participants in the morning. Urine specimens were placed in Eppendorf tubes with boric acid (2 g/L) as a preservative, centrifuged immediately, and stored at 4°C [48]. Urinary AD7c-NTP levels were assayed using an enzyme-linked immunosorbent assay AD7c-NTP kit (Anqun Biological Technology Co. Ltd., Shenzhen, China) [15]. According to the manufacturer’s instructions, 100 μL of the sample to be tested was added to the corresponding well and incubated at 37°C for one hour, before undergoing five consecutive wash steps with phosphate-buffered saline (PBS), avoiding the creation of bubbles during the washing process. Next, a biotinylated rabbit anti-AD7c-NTP antibody was added, and the sample was incubated at 37°C for 30 min. After a further five washes with PBS, the samples were incubated in horseradish peroxidase-labeled avidin at 37°C for another 30 min. Samples were again washed in PBS, and 50 μL of chromogenic reagents A and B were added in turn and incubated at 37°C for 15 min, with five washes in PBS after each incubation. Finally, the reaction was stopped by adding 50 μL of sulfuric acid as a stop buffer. A microplate reader (Multiskan Spectrum, Thermo Fisher Scientific, Waltham, MA, USA) at 450 nm wavelength was used to measure the optical density value of each sample. According to the standard curve of recombinant human AD7c-NTP peptides, the urinary AD7c-NTP level was then calculated.

### Classification of demographic characteristics and blood biochemical indicators

The 2180 subjects were categorized into four age groups (Table 1): < 60 years, 60–69 years, 70–79 years, and >80 years of age. Education levels were divided into three groups: less than a high school diploma (< 12 years of education), a high school diploma (12 years of education), and college or higher (> 12 years of education). Subjects who were currently married were referred to as with spouse group, while subjects who were currently unmarried, widowed, or divorced were referred to as without spouse group. MMSE scores ≤ 24 for subjects with more than six years of education were considered abnormal, and MMSE scores ≤ 20 for subjects with 1–6 years of education were considered abnormal. When the subject was illiterate, an MMSE score ≤ 17 was defined as abnormal [49].

The boundary values and groupings of blood biochemical indicators were as follows, based on the normal thresholds for each assay (Table 2). Male subjects with serum CR between 62 and 115 μmol/L were assigned to the normal group, while CR levels < 62 μmol/L were classified as reduced and CR levels > 115 μmol/L were classified as elevated. In female subjects, normal serum CR was 53–97 μmol/L, while < 53 μmol/L was considered reduced, and > 97 μmol/L was considered elevated. For UMA values, subjects with levels < 20 mg/L were classified as normal, and levels ≥ 20 mg/L were classified as the elevated group. In males, the normal value of UA was 200–420 μmol/L, while levels < 200 μmol/L were considered reduced, and levels > 420 μmol/L were considered elevated. In females, normal UA was classified as 140–340 μmol/L, while levels < 140 μmol/L were classified as reduced, and levels > 340 μmol/L were classified as elevated.

Subjects with GLU of 3.9–6.1 mmol/L were in the normal group, while levels < 3.9 mmol/L were classified as reduced, and levels > 6.1 mmol/L were considered elevated. The normal range of HbA1c was 4.0%–6.0%, while values > 6.0% were classified as elevated. The normal range of insulin was 2.6–24.9 μIU/mL, while levels < 2.6 μIU/mL were classified as reduced, and levels > 24.9 μIU/mL were considered elevated. Subjects with TC levels < 5.2 mmol/L were classified into the normal group, while levels ≥ 5.2mmol /L were considered as the elevated group. Subjects with TG < 1.7 mmol/L were considered normal and leveled ≥ 1.7 mmol/L were classified as the elevated group. For LDL-C measurements, values < 3.12 mmol/L were considered normal, while values ≥ 3.12 mmol/L were considered as the elevated group. Subjects with HDL-C > 0.91 mmol/L were assigned to the normal group, and levels ≤ 0.91 mmol/L were considered the reduced group. Subjects with hsCRP levels < 0.5 mg/dL were assigned to the normal group, while those with hsCRP ≥ 0.5 mg/dL were assigned to the elevated group.

### Statistical analysis

Statistical Package for Social Sciences (SPSS) 22.0 software was used to analyze all data. Data with continuous variables were expressed as the mean ± standard deviation (SD) if the distributions were normal, or as median (interquartile range) if the distributions were not normal. The two-independent-sample *t*-test was used to compare data between two groups, such as for sex, marital status, MMSE scores, and UMA, TC, TG, LDL-C, HDL-C, and hsCRP levels. The analysis of variance (ANOVA) was used to compare data among three or four groups, such as for age and years of education. The Mann–Whitney *U* test was used to compare data for variables with non-normal distributions, such as urinary AD7c-NTP and blood biochemical indicators. The chi-squared test was used to compare counting data when appropriate. Spearman’s correlation analysis was used to further analyze the correlation between blood biochemical indicators and urinary AD7c-NTP levels. The distribution of AD7c-NTP was fitted to normal after lognormal transformation and the adjusted age and sex AD7c-NTP levels were then estimated using general linear models. The threshold for statistical significance was set to *p* < 0.05. Participants with missing data were not included in the corresponding statistical analysis.

## Supplementary Material

Supplementary Figures
