# Supplementary material for: The relationship between urinary Alzheimer-associated neuronal thread protein and blood biochemical indicators in the general population
Source: Aging (Albany NY). 2020 Jul 31;12(15):15260–80. doi: 10.18632/aging.103356 (PMC7467383; doi:10.18632/aging.103356)
Supplement: Supplementary Figures [file aging-12-103356-s001..pdf]

SUPPLEMENTARY FIGURES

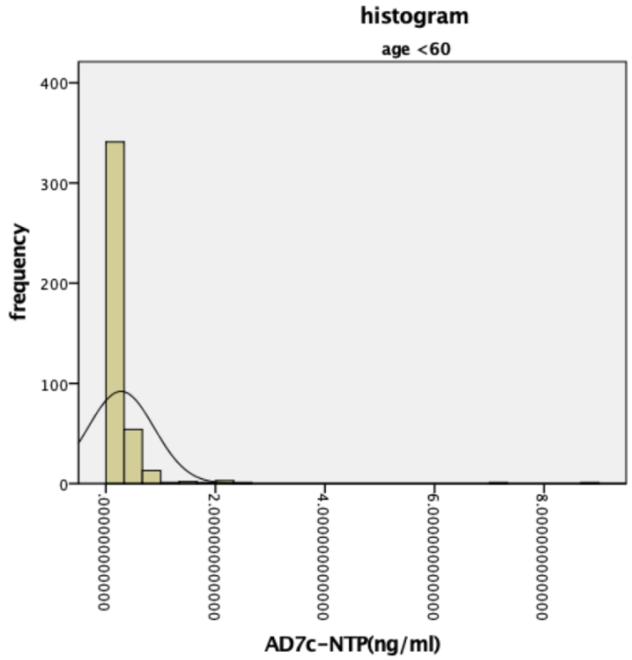

Supplementary Figure 1. The distribution and the histogram of urinary Alzheimer-associated neuronal thread protein (AD7c-NTP) in <60 years group.

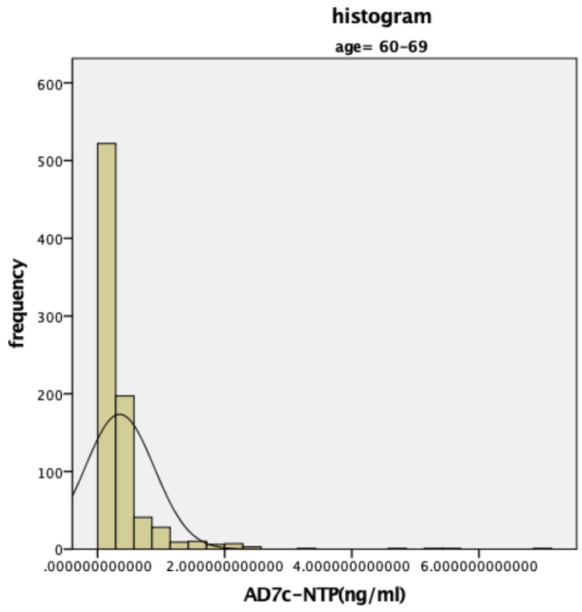

Supplementary Figure 2. The distribution and the histogram of urinary AD7c-NTP in 60-69 years group.

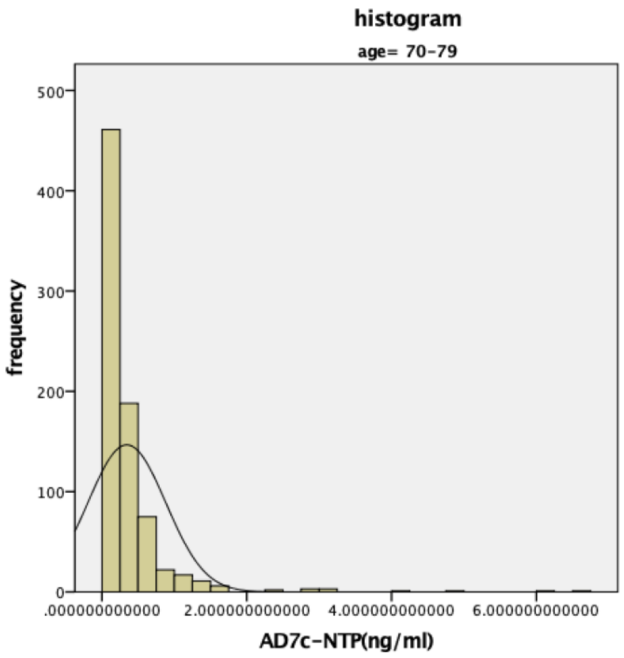

Supplementary Figure 3. The distribution and the histogram of urinary AD7c-NTP in 70-79 years group.

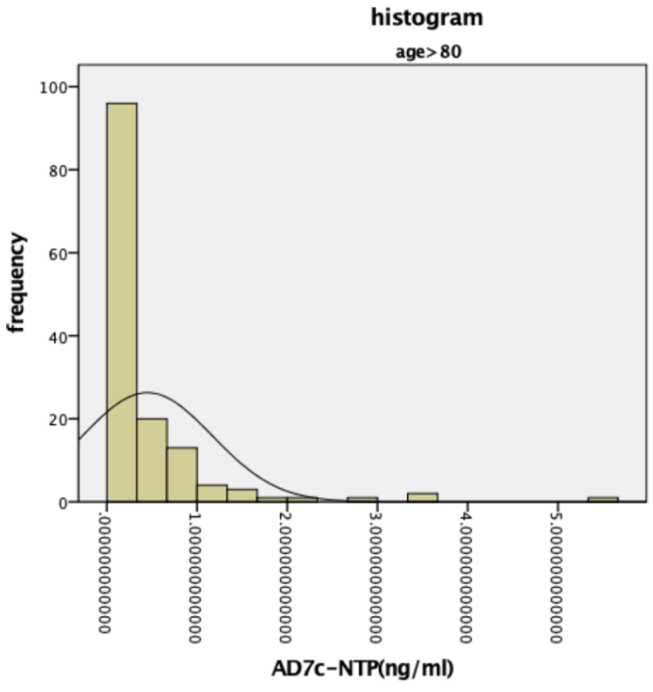

Supplementary Figure 4. The distribution and the histogram of urinary AD7c-NTP in >80 years group.

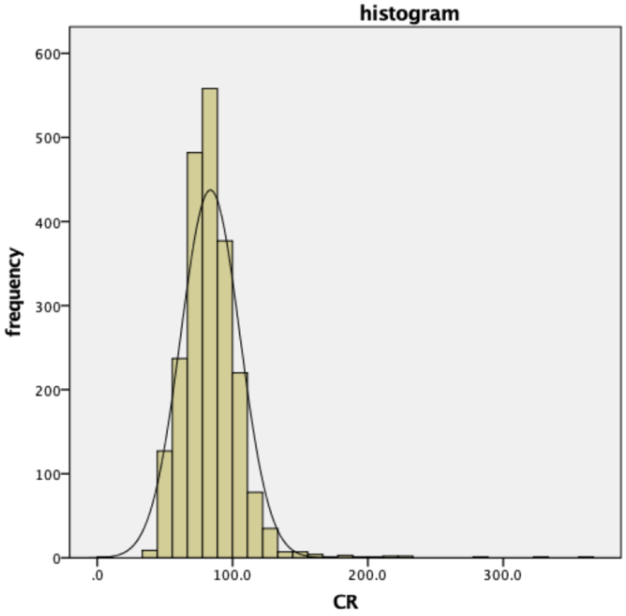

Supplementary Figure 5. The distribution and the histogram of serum creatinine (CR).

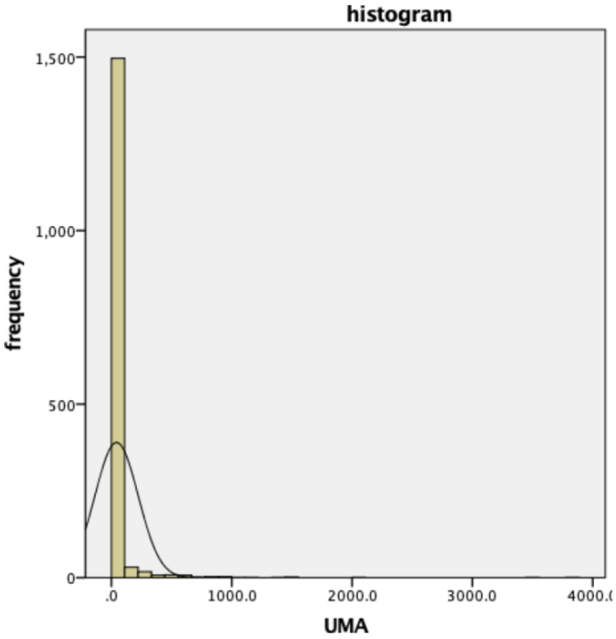

Supplementary Figure 6. The distribution and the histogram of urinary microalbumin (UMA).

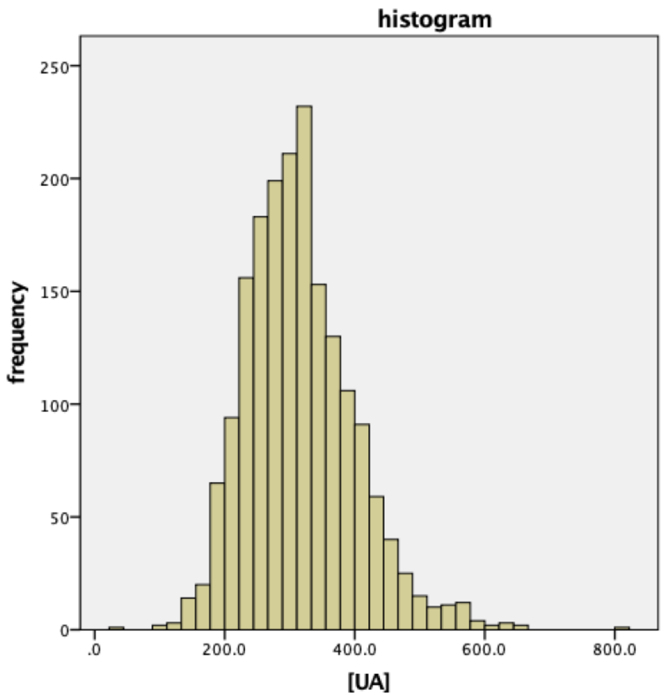

Supplementary Figure 7. The distribution and the histogram of uric acid (UA).

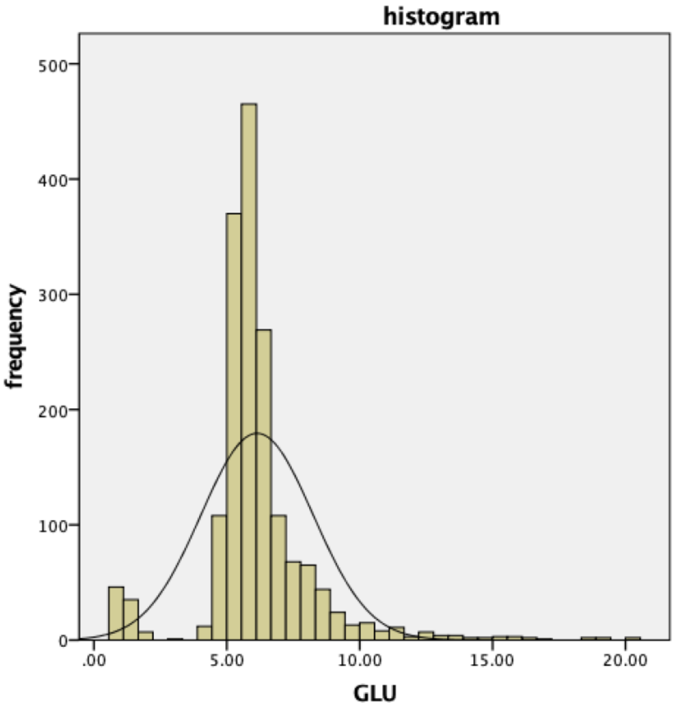

Supplementary Figure 8. The distribution and the histogram of glucose (GLU).

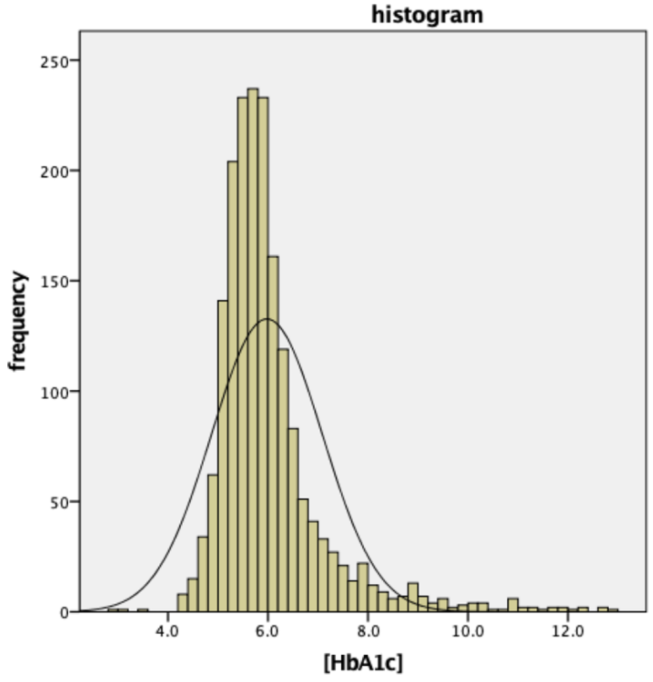

Supplementary Figure 9. The distribution and the histogram of hemoglobin A1c (HbA1c).

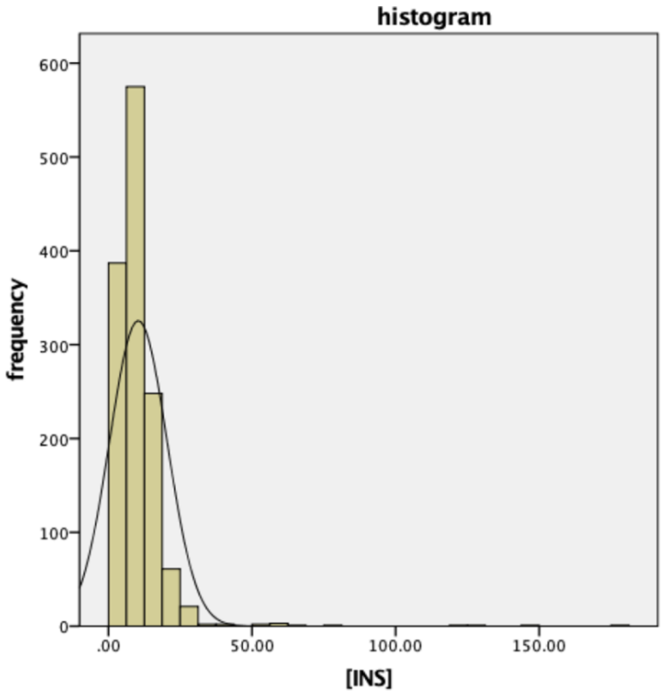

Supplementary Figure 10. The distribution and the histogram of insulin (INS).

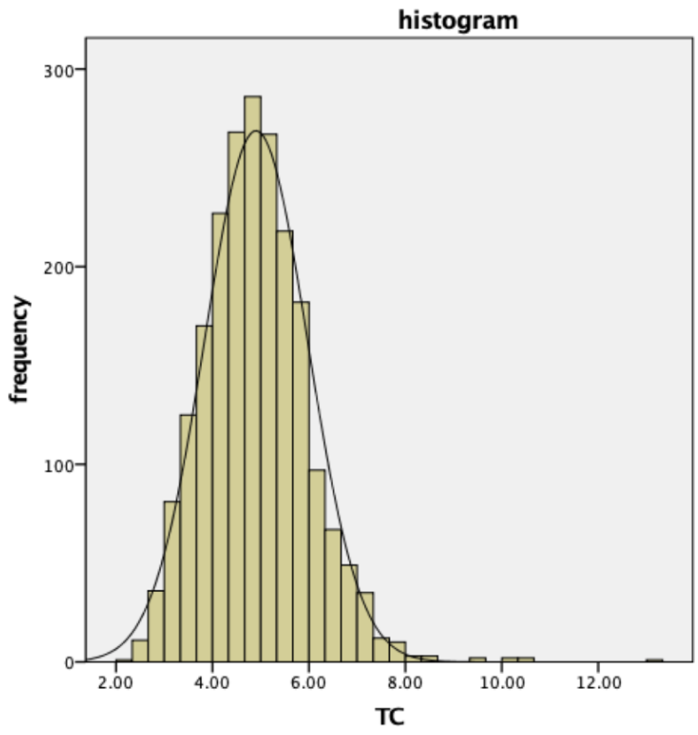

Supplementary Figure 11. The distribution and the histogram of total cholesterol (TC).

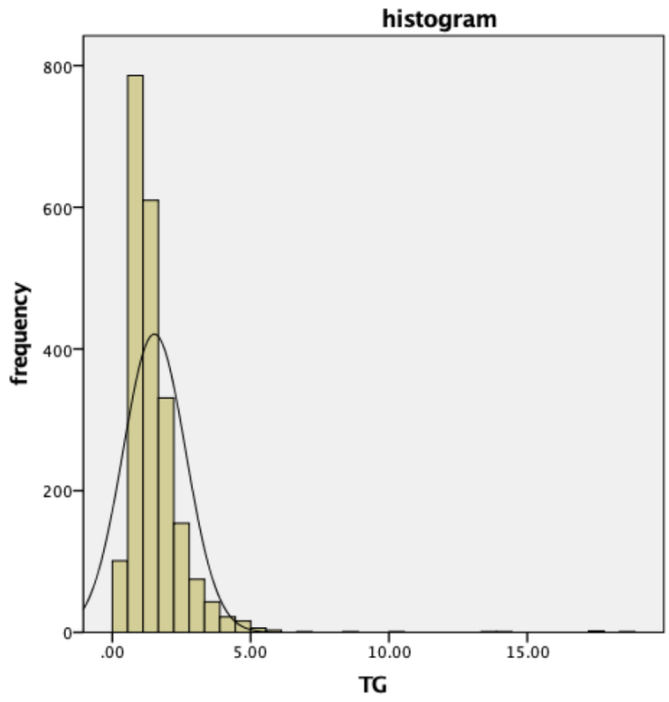

Supplementary Figure 12. The distribution and the histogram of triglycerides (TG).

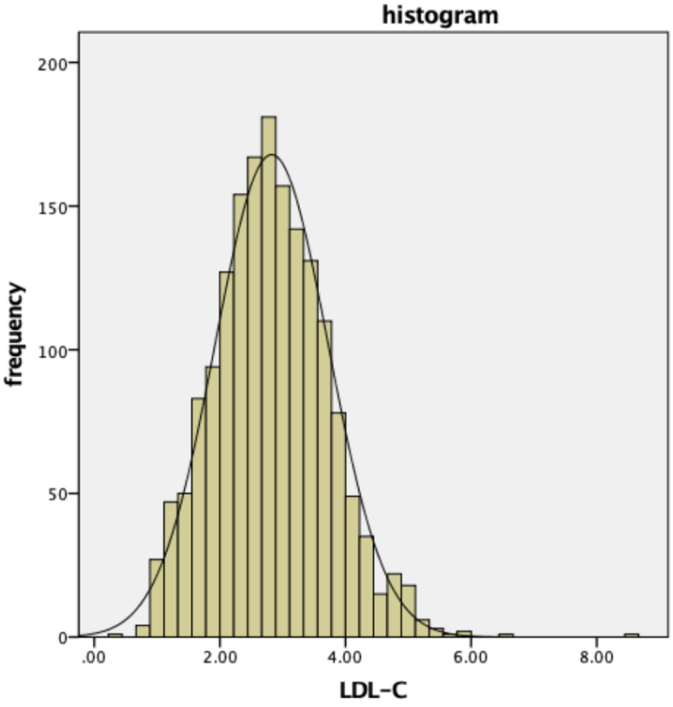

Supplementary Figure 13. The distribution and the histogram of low-density lipoprotein cholesterol (LDL-C).

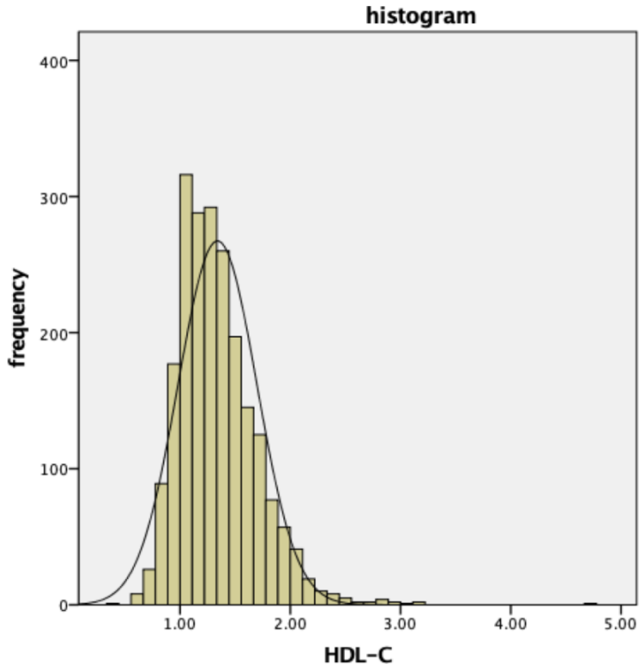

Supplementary Figure 14. The distribution and the histogram of high-density lipoprotein cholesterol (HDL-C).

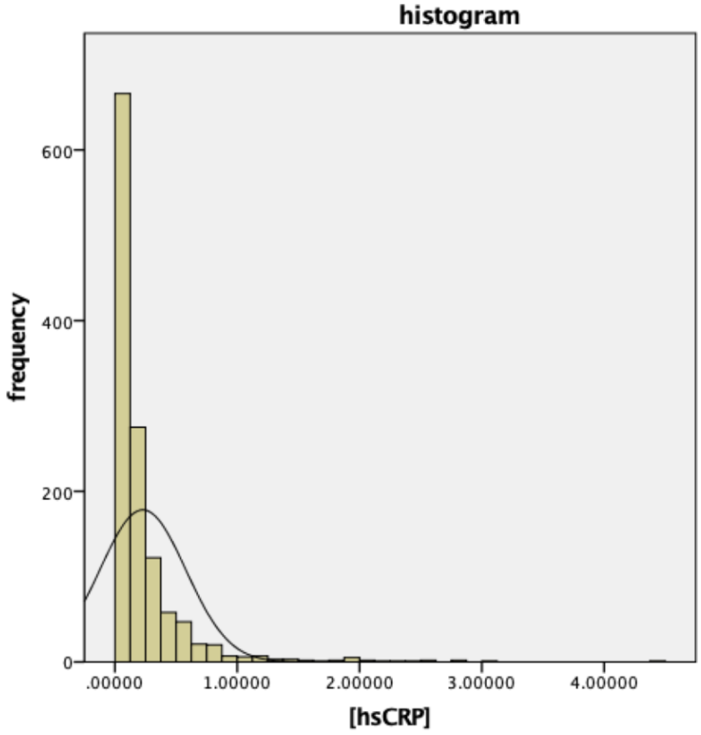

Supplementary Figure 15. The distribution and the histogram of hypersensitive C-reactive protein (hsCRP).

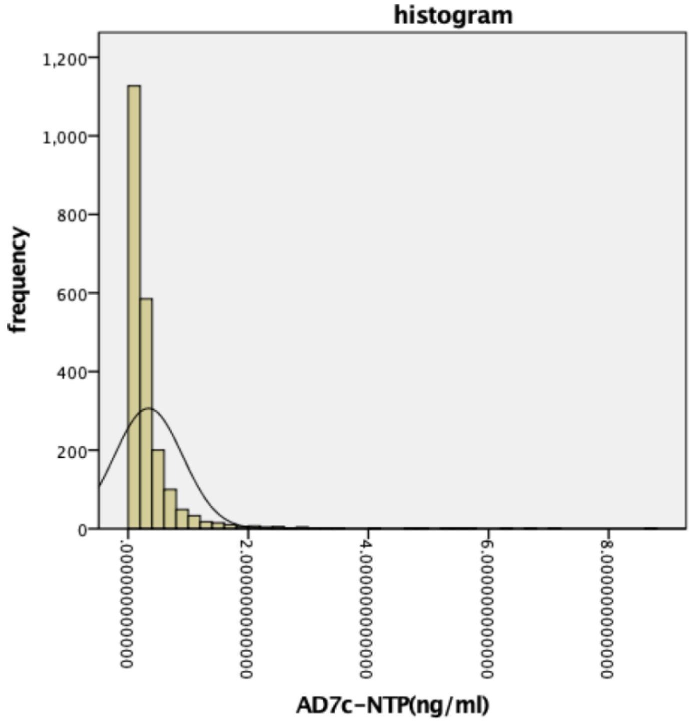

Supplementary Figure 16. The distribution and the histogram of urinary AD7c-NTP.
